# The Italian data on SARS-CoV-2 infection in transplanted patients support an organ specific immune response in liver recipients

**DOI:** 10.3389/fimmu.2023.1203854

**Published:** 2023-07-03

**Authors:** Maria Rendina, Michele Barone, Chiara Lillo, Silvia Trapani, Lucia Masiero, Paolo Trerotoli, Francesca Puoti, Luigi Giovanni Lupo, Francesco Tandoi, Salvatore Agnes, Antonio Grieco, Enzo Andorno, Simona Marenco, Edoardo Giovanni Giannini, Umberto Baccarani, Pierluigi Toniutto, Amedeo Carraro, Antonio Colecchia, Matteo Cescon, Maria Cristina Morelli, Umberto Cillo, Patrizia Burra, Paolo Angeli, Michele Colledan, Stefano Fagiuoli, Luciano De Carlis, Luca Belli, Paolo De Simone, Paola Carrai, Fabrizio Di Benedetto, Nicola De Maria, Giuseppe Maria Ettorre, Valerio Giannelli, Salvatore Gruttadauria, Riccardo Volpes, Sveva Corsale, Vincenzo Mazzaferro, Sherrie Bhoori, Renato Romagnoli, Silvia Martini, Giorgio Rossi, Lucio Caccamo, Maria Francesca Donato, Massimo Rossi, Stefano Ginanni Corradini, Marco Spada, Giuseppe Maggiore, Giuseppe Tisone, Ilaria Lenci, Giovanni Vennarecci, Raffaella Tortora, Marco Vivarelli, Gianluca Svegliati Baroni, Fausto Zamboni, Laura Mameli, Silvio Tafuri, Simona Simone, Loreto Gesualdo, Massimo Cardillo, Alfredo Di Leo

**Affiliations:** ^1^ Gastroenterology Unit, University Hospital Policlinico of Bari, Bari, Italy; ^2^ Gastroenterology Unit, Department of Precision and Regenerative Medicine - Ionian Area-, University of Bari Aldo Moro, Bari, Italy; ^3^ Italian National Transplant Center, National Institute of Health, Rome, Italy; ^4^ Section of Statistics, Interdisciplinary Department of Medicine, University of Bari Aldo Moro, Bari, Italy; ^5^ General Surgery and Liver Transplantation Unit, Department of Precision and Regenerative Medicine - Ionian Area-, University of Bari, Bari, Italy; ^6^ U.O.C. Chirurgia Generale e Trapianti di Organo, Policlinico Gemelli, Università Cattolica del Sacro Cuore, Roma, Italy; ^7^ U.O.C. Medicina Interna e del Trapianto di Fegato, Policlinico Gemelli, Università Cattolica del Sacro Cuore, Roma, Italy; ^8^ Chirurgia dei Trapianti di Fegato, IRCCS Ospedale Policlinico San Martino, University of Genoa, Genoa, Italy; ^9^ Gastroenterology Unit, IRCCS Ospedale Policlinico San Martino, University of Genoa, Genoa, Italy; ^10^ Centro Trapianto di Fegato, A.O.U.I. Udine, Università degli Studi di Udine, Udine, Italy; ^11^ U.S.D. Epatologia e Trapianto di Fegato, A.O.U.I. Udine, Università degli Studi di Udine, Udine, Italy; ^12^ U.S.D. Trapianti Epatici A.O.U.I. Verona, Verona, Italy; ^13^ Gastroenterology Unit, Department of Medical Specialties, University Hospital of Modena, Modena, Italy; ^14^ Chirurgia Epatobiliare e dei Trapianti, IRCCS, A.O.U. di Bologna, University of Bologna, Bologna, Italy; ^15^ Internal Medicine Unit for the Treatment of Severe Organ Failure, IRCCS, A.O.U. di Bologna, Bologna, Italy; ^16^ Hepatobiliary Surgery and Liver Transplantation, University-Teaching Hospital of Padova, Padova, Italy; ^17^ Multivisceral Transplant Unit, University-Teaching Hospital of Padova, Padova, Italy; ^18^ Unit of Internal Medicine and Hepatology (UIMH), University-Teaching Hospital of Padova, Padova, Italy; ^19^ U.O.C. Chirurgia Generale III, Centro Trapianti Fegato, A.S.S.T. Ospedale Papa Giovanni XXIII, Bergamo, Italy; ^20^ Gastroenterology Hepatology and Transplantation Unit, A.S.S.T. Ospedale Papa Giovanni XXIII, Bergamo, Italy; ^21^ Gastroenterologia, Department of Medicine University of Milan Bicocca, Milano, Italy; ^22^ Chirurgia Generale dei Trapianti, Azienda Ospedaliera Niguarda Ca’Granda, University of Milano-Bicocca, Milano, Italy; ^23^ U.O.C. Epatologia e Gastroenterologia, Azienda Ospedaliera Niguarda Ca’Granda, Milano, Italy; ^24^ U.O.C. Chirurgia Epatica e Trapianti di Fegato, A.O.U. Pisana, University of Pisa, Pisa, Italy; ^25^ U.O. Chirurgia Epatica e del Trapianto di Fegato, A.O.U. Pisana, Pisa, Italy; ^26^ U.O.C. di Chirurgia Oncologica Epatobiliopancreatica e Chirurgia dei Trapianti di Fegato, Azienda Ospedaliera Policlinico, Università di Modena, Modena, Italy; ^27^ U.O.C. Chirurgia Generale e Trapianti, Azienda Ospedaliera San Camillo Forlanini, Roma, Italy; ^28^ Hepatology Unit, Azienda Ospedaliera San Camillo Forlanini, Roma, Italy; ^29^ IRCCS-ISMETT-UPMCI, Palermo, Italy; ^30^ University of Catania, Catania, Italy; ^31^ Unità di Gastroenterologia ed Epatologia, IRCCS-ISMETT-UPMCI, Palermo, Italy; ^32^ Hepato-pancreatic-biliary surgery and Liver transplantation, Fondazione IRCCS Istituto Nazionale Tumori, Milan, Italy; ^33^ Department of Oncology, University of Milan, Milan, Italy; ^34^ Chirurgia Generale 2, Centro Trapianto Fegato A.O.U Città della Salute e della Scienza di Torino, Presidio Molinette, Torino, Italy; ^35^ Gastroenteroly Unit, A.O.U Città della Salute e della Scienza di Torino, Presidio Molinette, Torino, Italy; ^36^ Division of General and Liver Transplant Surgery, Ospedale Maggiore Policlinico, Milano, Italy; ^37^ Division of Gastroenterology and Hepatology, Ospedale Maggiore Policlinico, Milano, Italy; ^38^ U.O.C. di Chirurgia Generale e Trapianti di Organo, Policlinico Umberto I, Sapienza Università di Roma, Rome, Italy; ^39^ Gastroenterology Unit, Policlinico Umberto I, Sapienza Università di Roma, Rome, Italy; ^40^ Division of Hepatobiliopancreatic Surgery, Liver and Kidney Transplantation, Bambino Gesù Children’s Hospital, IRCCS, Rome, Italy; ^41^ Hepatogastroenterology, Digestive Endoscopy, Nutrition and Liver Transplantation Unit, Gesù Children’s Hospital, IRCCS, Rome, Italy; ^42^ Liver Transplant Unit, A.O.U. Policlinico Tor Vergata, University of Tor Vergata Rome, Rome, Italy; ^43^ Hepatology Unit, A.O.U. Policlinico Tor Vergata, University of Tor Vergata Rome, Rome, Italy; ^44^ Hepatobiliary and Liver Tranplantation Surgery, A.O.R.N. “A. CARDARELLI”, Naples, Italy; ^45^ Liver Unit, A.O.R.N. “A. CARDARELLI”, Naples, Italy; ^46^ Chirurgia Epatobiliare, Pancreatica e dei Trapianti, A.O.U., Ospedali Riuniti, Ancona, Italy; ^47^ Gastroenterology Unit, A.O.U., Ospedali Riuniti, Ancona, Italy; ^48^ General and Hepatic Transplantation Surgery Unit, AO.B. G. Brotzu, Cagliari, Italy; ^49^ Interdisciplinary Department of Medicine, University of Bari Aldo Moro, Bari, Italy; ^50^ Dialysis and Kidney Transplantation Unit, Department of Precision and Regenerative Medicine - Ionian Area-, University of Bari Aldo Moro, Bari, Italy

**Keywords:** COVID-19, liver transplantation, solid organ transplant, immunotolerance, microchimerism

## Abstract

**Introduction:**

The study of immune response to SARSCoV-2 infection in different solid organ transplant settings represents an opportunity for clarifying the interplay between SARS-CoV-2 and the immune system. In our nationwide registry study from Italy, we specifically evaluated, during the first wave pandemic, i.e., in non-vaccinated patients, COVID-19 prevalence of infection, mortality, and lethality in liver transplant recipients (LTRs), using non-liver solid transplant recipients (NL-SOTRs) and the Italian general population (GP) as comparators.

**Methods:**

Case collection started from February 21 to June 22, 2020, using the data from the National Institute of Health and National Transplant Center, whereas the data analysis was performed on September 30, 2020.To compare the sex- and age-adjusted distribution of infection, mortality, and lethality in LTRs, NL-SOTRs, and Italian GP we applied an indirect standardization method to determine the standardized rate.

**Results:**

Among the 43,983 Italian SOTRs with a functioning graft, LTRs accounted for 14,168 patients, of whom 89 were SARS-CoV-2 infected. In the 29,815 NL-SOTRs, 361 cases of SARS-CoV-2 infection were observed. The geographical distribution of the disease was highly variable across the different Italian regions. The standardized rate of infection, mortality, and lethality rates in LTRs resulted lower compared to NL-SOTRs [1.02 (95%CI 0.81-1.23) vs. 2.01 (95%CI 1.8-2.2); 1.0 (95%CI 0.5-1.5) vs. 4.5 (95%CI 3.6-5.3); 1.6 (95%CI 0.7-2.4) vs. 2.8 (95%CI 2.2-3.3), respectively] and comparable to the Italian GP.

**Discussion:**

According to the most recent studies on SOTRs and SARS-CoV-2 infection, our data strongly suggest that, in contrast to what was observed in NL-SOTRs receiving a similar immunosuppressive therapy, LTRs have the same risk of SARS-CoV-2 infection, mortality, and lethality observed in the general population. These results suggest an immune response to SARS-CoV-2 infection in LTRS that is different from NL-SOTRs, probably related to the ability of the grafted liver to induce immunotolerance.

## 1 Introduction

Since it began, the COVID-19 pandemic has been considered a matter of great concern in the transplant setting due to the high expected increased risk of infection, adverse outcomes, and death. This alarming perception was related to the chronic use of immunosuppressive drugs associated with comorbidities (diabetes, obesity, hypertension, older age), which are frequent negative predictive factors for COVID-19 outcomes (1–4).

In the absence of population-based epidemiological surveillance, it was hard to determine the true incidence of SARS-CoV-2 in all clinical settings, especially during the initial first wave of the pandemic (5, 6). However, the impact of SARSCoV-2 infection in a specific sub-population, such as the pharmacologically immunosuppressed solid organ transplant recipients (SOTRs), represents an occasion for clarifying the interplay between SARS-CoV-2 and the immune system (7).

Regarding liver transplant recipients (LTRs), the currently available data have reached different conclusions on incidence and mortality (8–14). Italy was the COVID-19 epicenter during the first wave of the pandemic, thus representing an interesting model for studying the transplant setting without the interference of possible therapies and vaccination (15–17). At that time, a nationwide population-based study by Trapani et al. (18) showed that SOTRs, all together, experienced a higher cumulative incidence of SARS-CoV-2 infection and a double 60-day case fatality as compared to the general population (GP) (1.02% vs. 0.4%, p< 0.05; 30.6% vs. 15.4%, respectively). However, they observed a gradient through the different organs.

In our nationwide registry study from Italy, we specifically evaluated, during the first wave pandemic, if SARS-CoV-2 infection significantly influenced the standardized age and sex prevalence of infection, mortality, and lethality in LTRs using non-liver SOTRs (NL-SOTRs) and Italian GP as comparators.

## 2 Patients and methods

### 2.1 Data collection

This study was based on a sub-analysis of the nationwide population-based study by Trapani et al. (18). Data on all cases of laboratory-confirmed SARS-CoV-2 positivity in Italy were collected by the National Institute of Health (Istituto Superiore di Sanità-ISS), which was appointed as the coordinator of the COVID-19 surveillance system with the task of gathering microbiological and epidemiological data provided daily by the Italian Regions and Autonomous Provinces (Regions) (19). Data on all laboratory confirmed COVID+ cases per definitions published and regularly updated by the European Centre for Disease Prevention and Control (ECDC) were collected in the system (20). All the data on SOTRs were collected prospectively and analyzed retrospectively through the Information Transplant System (SIT), which is managed by National Transplant Centre. National COVID+ cases, were cross-referenced with SOTRs as recorded in the SIT. The record linkage between the two databases was implemented through deterministic technique, thanks to the presence of personal data for all patients identifying the unique correspondence between patients from the two databases. The case collection time in the present study started from February 21 to June 22, 2020, and the data analysis was performed on December 27, 2020, to consolidate data reporting.

### 2.2 Risk of SARS-CoV-2 infection and lethality in Italian LTRs

To assess standardized rates of SARS-CoV-2 infection in Italian LTRs compared to NL-SOTRs (kidney, heart, lung, and pancreas) and the general population, we defined the following groups: a) Italian general population (GP; n= 59,497,002 as per ISTAT data of January 1, 2020), b) solid organ transplant recipients (NL-SOTRs; n= 29,815), and c) liver transplant recipients (LTRs; n= 14,168). Kidney, heart, lung, and pancreas accounted for 83.1%, 11.3%, 3.6%, and 2.7% of NL-SOTRs in Italy (National Transplant Centre data, 2019). The study was conducted in accordance with the Declaration of Helsinki (2000) and was approved by the local Ethics Committee (N.237/DG).

### 2.3 Statistical analysis

Crude and specific rates by sex and age of SARS-CoV-2 infection and mortality were determined as the rate between the number of infections or death (Italian Ministry of Health, 2020 Bulletin COVID-19 Outbreak in Italy) and the total number of subjects of each single population (GP, LTRs and NL-SOTRs) (ISTAT data of January 1, 2020). Crude and specific rate by sex and age of SARS-CoV-2 lethality was determined as the rate between the number of death cases and the SARS-CoV-2 positive cases in each population.

To compare distribution of infection, mortality, and lethality between the LTRs in respect to the Italian GP and NL-SOTRs population, adjusted by sex and age, we applied an indirect standardization method determining the standardized rate (SR). In detail, all three groups, LTRs, NL-SOTRs and GP, were stratified by sex (male and female) and age class (0-19 years, 20-49 years, 50-69 years and 70 and more years). The standardized rate of prevalence and its corresponding 95% confidence interval were obtained by the ratio between observed cases and expected cases. The same procedure was applied for mortality and lethality. All analysis were performed by SAS/STAT^®^ Statistics version 9.4 (SAS Institute, Cary, NC, USA).

## 3 Results

### 3.1 General data on the three different populations

**Figure 1:**
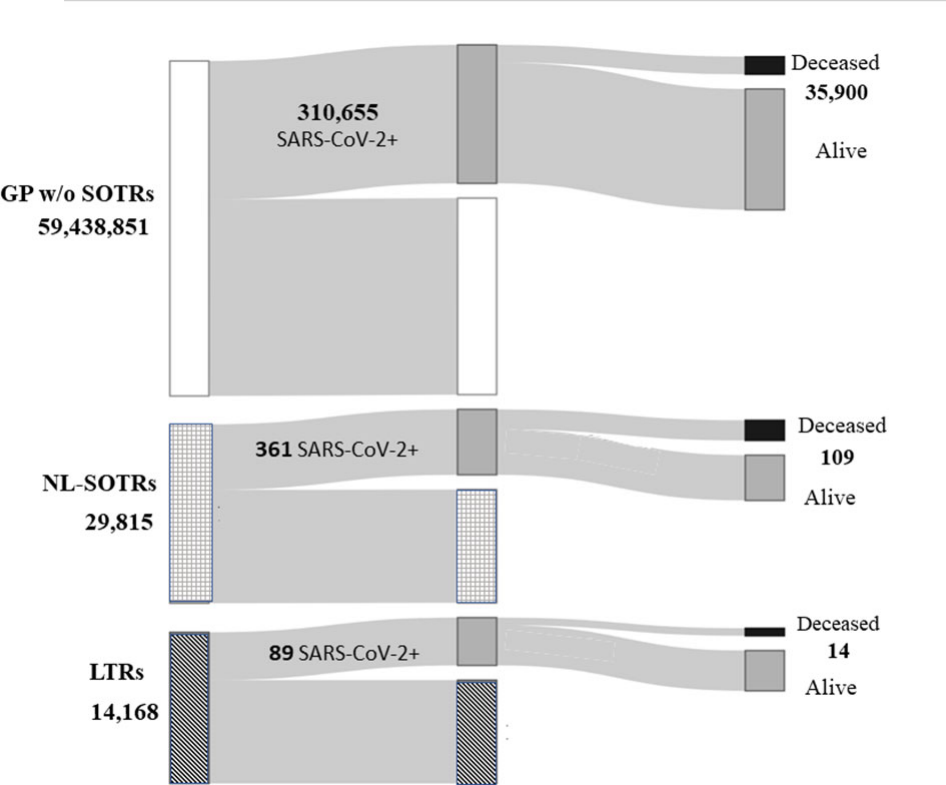
Total Italian general population, total non-liver solid organ transplant recipients and liver transplant recipients, and the number of patients with and without SARS-CoV-2 infection, with the corresponding number of deaths. GP, general population; NL-SOTRs, non-liver solid organ transplant recipients; LTRs, liver transplant recipients.

**Figure 1** describes the number of subjects composing the entire GP, NL-SOTRs, and LTRs population, as well as the number of patients with and without SARS-CoV-2 infection, with the corresponding number of deaths.

From February 21, when the first case of SARS-CoV-2 infection was detected in Italy, to June 22, 2020, there were 310,655 cases of infection in the whole Italian GP, with 35,900 deaths, while in NL-SOTRS and LTRs 361 and 89 cases of infection and 109 and 14 cases of death were observed, respectively (**Figure 1**).

### 3.2 Geographical distribution, cumulative incidence, and deaths

**Table 1** reports SARS-CoV-2 cumulative incidence and number of deaths of LTRs among SOTRs, according to geographical distribution across the different Italian regions.

**Table 1:** SARS-CoV-2 Cumulative Incidence and Death in LT population among SOTRs according to geographical distribution across Italian regions.

Regions	All Italian SOTRs	COVID+ among All SOTRs		All Italian LTRs	COVID+ Among LTRs		% LTRs/all SOTRs	Cumulative Incidence COVID+ among LTRs			Deaths among LTRs-COVID+	
	N	N	%	N	N	%	%	x100 LTRs	95% CI		N	%
**Italy**	43,983	450		14,168	89^	19.8	32.2	0.631	0.513	0.777	14^^	15.7
**Piedmont**	3,684	68	15.1	1,130	13	14.6	30.7	1.159	0.673	1.995	2	
**Valled’Aosta**	135	3	0.7	46			34.1					
**Lombardy**	7,810	233	51.8	2,610	51	57.3	33.4	1.965	1.493	2.585	10	
**PABZ**	77	1	0.2	20			26					
**PATN**	335	5	1.1	84			25.1					
**Veneto**	3,583	30	6.7	856	5	5.6	23.9	0.588	0.245	1.412	1	
**Friuli Venezia G.**	990	0	0	258			26.1					
**Liguria**	1,239	15	3.3	390	4	4.5	31.5	1.026	0.385	2.733	1	
**Emilia Romagna**	3,124	36	8	1,024	5	5.6	32.8	0.49	0.204	1.177		
**Tuscany**	2,684	11	2.4	1,043	3	3.4	38.9	0.289	0.093	0.896		
**Umbria**	539	3	0.7	166	2	2.2	30.8	1.205	0.301	4.817		
**Marche**	1,011	12	2.7	317	3	3.4	31.4	0.946	0.305	2.934		
**Lazio**	3,881	8	1.8	1,213	2	2.2	31.3	0.167	0.042	0.666		
**Abruzzo**	826	3	0.7	218			26.4					
**Molise**	228	0	0	69			30.3					
**Campania**	4,571	8	1.8	1,829	1	1.1	40	0.055	0.008	0.389		
**Puglia**	2,824	6	1.3	798			28.3					
**Basilicata**	338	0	0	115			34					
**Calabria**	1,260	0	0	410			32.5					
**Sicilia**	3,271	4	0.9	915			28					
**Sardegna**	1,272	4	0.9	464			36.5					
**Foreign State**	302			193			63.9					

CI, cumulative incidence; COVID*, SARS-COV-2 positive; IQR, interquartile range; SOTRs, solid organ transplant recipients; LTRs, liver transplant recipients.

^ Including 2 combined liver-kidney transplant (1 in Lumbardy and 1 in Piedmont). ^^ Any combined liver-kidney transplant(a) CI. Cumulative Incidence of COVID + LTRs with reference to all Italian SOTRs. *Trapani S.Masiero L.Puoti F.et al. Incidence and outcome of SARS-CoV-2 infection on solid organ transplantation recipients: A nationwide population-based study. Am J Transplant. 2021;21:2509-2521. doi:10.1111/ajt.16428 (20).

Among the 43,983 Italian SOTRs with a functioning graft, LTRs accounted for 14,168 patients, with a regional average of LTRs/SOTRs of 32.2% (**Table 1**). Among 14,168 LTRs, 89 were SARS-CoV-2 infected during the interval period. The cumulative 95%CI of infection ranged broadly at the regional level, from a minimum of 0.05% [95% CI 0.008-0.39] in Campania (1 case), to a maximum value of 1.95% in Lombardy [95% CI1.49-2.58] with 51 cases (**Table 1**). The number of SARS-CoV2 infections in LTRs was 89 (68 males, 21 females), so the prevalence for this group was 62.8 per 10,000 inhabitants; in NL-SOTRs, 361 cases (272 males, 89 females) were observed with a prevalence of 121.9 per 10,000 inhabitants, while in GP the number of cases was 310,655, with a prevalence of 52.3 per 10,000 inhabitants.

### 3.3 Crude and adjusted infection, mortality, and lethality rates

**Table 2** shows the specific sex and age class rate of infection, mortality and lethality in the three groups. The effect of age was more evident in the older age class (≥ 70 years), where the rate was about 97 per 10,000 in both sexes in GP, while in LTRs it was 43.6 for males and 38.0 for females; on the other hand, the rates for NL-SOTRs were higher compared to the previous two groups in the last age class: 132.7 per 10,000 in male and 104.5 per 10,000 in female. Most importantly, the standardized rate of infection (SRI) in LTRs was 1.02 (95%CI 0.81 to 1.23), whereas in NL-SOTRs it was 2.01 (95% CI 1.8-2.2) compared to GP, meaning that in NL-SOTRs the prevalence of infection was twice that encountered in GP and LTRs. The total number of deaths (**Table 2**) was 35,900 (20,581 males, 15,319 females) in GP, 14 (13 males, 1 female) in LTRs, and 109 (85 males, 24 females) in NL-SOTRs, with a crude mortality rate of 6.0, 9.9, and 36.8 per 10,000 inhabitants, respectively. In these three populations, a higher mortality rate was observed in the older age class. In detail, the mortality rates were 37.6 and 23.3 per 10,000 for males and females, respectively, in the GP group, while in LTRs the rates were lower: 17.5 and 12.9 per 10,000 for males and females, respectively; on the other hand, the specific mortality rates in NL-SOTRs were 79.1 per 10,000 and 44.0 per 10,000 for males and females, respectively. The standardized mortality rate was 1.0 (95% CI 0.5 to 1.5) in LTRs and 4.5 (95% CI 3.6-5.3) in NL-SOTRs, meaning that the risk of death in the latter group was more than four times higher respect to both GP and LTRs.

**Table 2:** Number of registered populations, cases of SARS-CoV2 positivity and deaths related to COVID-19 in the year 2020 by Italian and transplants populations.

	Age class	MALE					FEMALE				
		Positive to SARS-CoV-2	Prevalence x 10,000	Death for COVID-19	Mortality rate x 10,000	Lethality rate x 100	Positive to SARS-CoV-2	Prevalence x 10,000	Death for COVID-19	Mortality rate x 10,000	Lethality rate x 100
Italian Population	00-19	9,089	16.7	1	0.00	0.01	8,122	15.9	3	0.01	0.04
	20-49	48,722	44.5	287	0.26	0.59	52,106	48.7	116	0.11	0.22
	50-69	47,326	57.9	3,689	4.52	7.79	43,779	50.6	1,165	1.35	2.66
	70+	43,117	97.6	16,604	37.60	38.51	58,394	97.1	14,035	23.35	24.04
	Total	148,254	51.2	20,581	7.11	13.88	162,401	53.3	15,319	5.03	9.43
Liver transplant population (LTRs)	00-19	2	48.0		0.00	0.00	1	23.7		0.00	0.00
	20-49	5	56.7		0.00	0.00	3	43.3		0.00	0.00
	50-69	51	75.3	9	13.29	17.65	14	73.2		0.00	0.00
	70+	10	43.6	4	17.45	40.00	3	38.6	1	12.87	33.33
	Total	68	65.6	13	12.54	19.12	21	55.2	1	2.63	4.76
Non-Liver Solid Organ Transplant Population (NL-LTRs)	00-19	1	25.1		0.00	0.00	1	36.6		0.00	0.00
	20-49	54	109.5	5	10.14	9.26	13	42.7	1	3.28	7.69
	50-69	165	168.1	49	49.92	29.70	56	103.4	15	27.69	26.79
	70+	52	132.7	31	79.12	59.62	19	104.5	8	44.00	42.11
	Total	272	142.7	85	44.59	31.25	89	84.3	24	22.74	26.97

The crude rate of lethality was 15.7% (14 death/89 infected) in the LTRs, 11.6% (35,900/310,655) in GP, and 30.2% (109/361) in NL-SOTRs (**Table 2**). When we evaluated the sex- and age-adjusted lethality rates in the ≥ 70-year class, they were similar in GP and LTRs (38.5% for males and 24% for females, and 40% and 33%, respectively). Interestingly, the lethality rates in the NL-SOTRs population were higher in all age classes reaching 59.6% in males and 42.1% in females. The lethality (SR) was 1.6 (95% CI 0.7-2.4) in LTRs and 2.8 (95% CI 2.2-3.3) in NL-SOTRs, thus confirming a higher risk of death in NL-SOTRs compared to LTRs.

## 4 Discussion

### 4.1 Clinical and epidemiological data

Solid organ transplant patients have been identified as a group at higher risk of SARS-CoV-2 infection (9, 11, 18, 21, 22) and being immunosuppressed, exposed to more adverse outcomes than non-transplanted subjects (23). Theoretically, chronic immunosuppression and its association with multiple comorbidities (older age, diabetes, obesity, and hypertension) could account for this (1, 23).

In the present study, we further analyzed the favorable trend previously observed (18), showing that, among COVID-19+ SOTRs, those who underwent liver transplants had a significantly lower risk of infection, mortality, and lethality compared to NL-SOTRs. Moreover, we suggested the hypothesis that the outcomes similar to the general population could depend on the interplay between the liver and the “downregulated” immune system (see later “the tolerogenic hypothesis”).

Preliminary reports on SARS-CoV-2 susceptibility and outcome in liver transplantation were mainly based on uncontrolled case series reaching opposite conclusions, in favor of or against a better outcome (9–11, 17, 18, 24, 25).

Altogether this complex scenario is a tricky matter in immunosuppressed patients. Confounding factors could be related to the immunosuppressive status, which could play a role in mitigating symptoms or favoring the escape from testing criteria (21); on the other hand, Guarino et al. recently showed that LTRs are more frequently symptomatic than the general population (53.3% vs. 15.8%, p< 0.000), thus increasing the probability to register the infection (25). Chronic immunosuppression, particularly calcineurin inhibitors, could favor the early phase of viral replication, favoring the immune activation leading to a cytokine storm, responsible for the severe form of COVID-19; at the same time, these drugs could mitigate the cytokine storm by reducing immunoreactivity (26, 27). Belli et al. (28) found that the use of tacrolimus, i.e., the cornerstone of immunosuppression in the transplant setting, has a beneficial effect on SARSCoV-2 infections and was associated with a better survival rate in the 243 adult LTRs analyzed in the ELITA-ELTR Multi-center European Study.

In our study, the results on mortality agree with recent studies showing that LTRs do not have an increased risk of mortality compared to the general population (29–32).

Regarding the SARS-CoV-2 risk of infection in LTRs, our data seems different from that of the prospective Nationwide study conducted in Spain. This study reports a higher standardized incidence of viral infection in LTRs compared to the matched GP (33), although it was realized during the same period. This discrepancy could be due to a different distribution of infection and mortality across the two nations (34, 35). As a novelty, we showed that an organ-transplant gradient could exist among SOTRs in susceptibility and mortality rate for SARS-CoV-2 infection, although they were exposed to the same risk of infection and complication.

The “intensity” of immunosuppressive therapy in LTRs and NL-SOTRs could be responsible for the different susceptibility and adverse outcomes of SARS-CoV-2 infections (36–44). The difference in the immunosuppression therapy between the liver and kidney transplant, used as a reference, relies prevalently on the use of thymoglobulin induction, tacrolimus levels, mycophenolate, and steroids. The good marriage between LT and SARS-CoV-2 infection was later confirmed by the higher rate of humoral response to vaccination reaching 50% of LTRs in the preliminary experience of Marion et al. (45) in respect to 30% average in NL-SOTRS. In a recent study comparing humoral and cellular vaccine responses to SARS-CoV-2 in KTRs and LTRs, the use of steroids and MMF was significantly higher in KTRs (78.8% vs. 32.0%; p< 0.001) (46). However, although in LTRs the reduced immunosuppression was associated with a higher antibody response against SARS-CoV-2 after vaccination, again the liver advantage persisted after adjusting for immunosuppression (46).

### 4.2 The tolerogenic hypothesis

Another explanation for the favorable SARS-CoV-2 outcome in LTRs comes from the existence of an interaction between the downregulation of the immune system by the anti-rejection therapy and the tolerogenic activity of the liver (tolerogenic hypothesis). The liver has a proper immune system consisting of multiple lines of liver resident immune cells that can modulate immune tolerance (46–49), as supported by several lines of evidence: 1) the induction of oral tolerance to food antigens; 2) the spontaneous acceptance of liver grafts occurring in several experimental models; 3) the possibility to wean off anti-rejection therapies in about a quarter of clinical transplant recipients; 4) the capability of the liver to extend tolerance to other grafts such as kidney, despite major histocompatibility complex (MHC) mismatch (47–55).But what makes the liver graft extremely peculiar from an immunologic point of view is its unique ability to modulate the recipient’s immune system, which becomes tolerogenic through the migration of the multiple lines of liver resident immune cells from the engrafted liver into the recipient. Such a phenomenon, known as microchimerism, is the basis of the uniqueness of the liver immuno-transplant model compared to other types of solid organ transplants (52). This biological phenomenon, together with the clinical evidence of reduced mortality and lethality, could explain both the paucity of liver damage during COVID-19 (56, 57) and the difference in SARS-CoV-2 infection outcomes in LTRs compared to the other SOTRs (8, 22, 28, 30, 58, 59).

### 4.3 Limitations

This study presents some limitations. Firstly, it refers to the first epidemic wave, when several drawbacks arose in data reported by studies and health authority reports. Secondly, the most important limitation refers to the diagnostic criteria applied by healthcare systems in the world. These criteria have been not homogeneously applied in various countries and at the regional level; moreover, indications for testing varied greatly over weeks. However, as these biases apply to all populations studied, this mitigates the epidemiological limitations of our study. Thirdly, the low ratio between LTRs infected/total number LTRs could have influenced the incidence rate calculation; nevertheless, the great attention to the medical care in these patients consistently overcomes the underestimation of SARS-CoV-2 diagnosis. Fourthly, we did not report the data on immunosuppressive therapy of LTRs; however, data on the comparison between LTRs and KTRs show that the liver advantage persists after adjusting for immunosuppression. Finally, we did not compare the comorbidities in LTRs, NL-SOTRs. However, as reported by Trapani et al. (18), the multivariable logistic regression analysis on 30–60-day 95% CI of mortality conducted on all COVID-19+ patients revealed that only organ transplantation was the variable independently associated with mortality.

### 4.4 Conclusions

In conclusion, our data, according to the most recent large studies on SOTRs and SARS-CoV-2 infection, strongly suggest that liver transplant is not associated with an increased risk of SARS-CoV-2 infection, mortality, and lethality compared to the general population, in contrast to what was observed in NL-SOTRs, supporting the evidence of the immunological peculiarity of the liver graft.

## Data availability statement

The raw data supporting the conclusions of this article will be made available by the authors, without undue reservation.

## Ethics statement

The studies involving human participants were reviewed and approved by Ethics Committee of Azienda Ospedaliero-Universitaria Policlinico. Written informed consent for participation was not required for this study in accordance with the national legislation and the institutional requirements.

## Author contributions

All listed authors contributed to the data concerning the epidemiology of COVID-19. They have updated daily, the National Institute of Health (Istituto Superiore di Sanità-ISS) starting from February 22, 2020, and the National Transplant Center. In addition, all of them read the manuscript, gave their contribution, and approved it. EG, SS, and LG mediated the collaboration with the National Institute of Health and the National Transplant Center.
